# Predicting sleep based on physical activity, light exposure, and Heart rate variability data using wearable devices

**DOI:** 10.1080/07853890.2024.2405077

**Published:** 2024-09-19

**Authors:** Kyung Mee Park, Sang Eun Lee, Changhee Lee, Hyun Duck Hwang, Do Hoon Yoon, Eunchae Choi, Eun Lee

**Affiliations:** a Department of Hospital Medicine, Yongin Severance Hospital, Yonsei University College of Medicine, Yongin, Republic of Korea; b Department of Psychiatry, Institute of Behavioral Science in Medicine, and Institute for Innovation in Digital Healthcare, Severance Hospital, Yonsei University College of Medicine, Seoul, Republic of Korea; c Health IT center, Yonsei University Health System, Yonsei College of Medicine, Seoul, Republic of Korea; d Department of Artificial Intelligence, Chung-Ang University, Seoul, Republic of Korea; e Institute for Innovation in Digital Healthcare, Yonsei University, Seoul, Republic of Korea

**Keywords:** Sleep, sleep prediction, actigraphy, wearable device, machine learning, deep learning

## Abstract

**Objective:**

We aimed to improve the performance of sleep prediction algorithms by increasing the data amount, adding variables reflecting psychological state, and adjusting the data length.

**Materials and Methods:**

We used ActiGraph GT3X+^®^ and Galaxy Watch Active2^™^ to collect physical activity and light exposure data. We collected heart rate variability (HRV) data with the Galaxy Watch. We evaluated the performance of sleep prediction algorithms based on different data sources (wearable devices only, sleep diary only, or both), data lengths (1, 2, or 3 days), and analysis methods. We defined the target outcome, ‘good sleep’, as ≥90% sleep efficiency.

**Results:**

Among 278 participants who denied having sleep disturbance, we used data including 2136 total days and nights from 230 participants. The performance of the sleep prediction algorithms improved with an increased amount of data and added HRV data. The model with the best performance was the extreme gradient boosting model; XGBoost, using both sources combined data with HRV, and 2-day data (accuracy=.85, area under the curve =.80).

**Conclusions:**

The results show that the performance of the sleep prediction models improved by increasing the data amount and adding HRV data. Further studies targeting insomnia patients and applied researches on non-pharmacological insomnia treatment are needed.

## Introduction

The digitalization of cognitive behavior therapy for insomnia (CBTi) has proven to be an irreversible trend as digital CBTi has become one of the most used digital therapeutics worldwide [1]. However, sleep diaries continue to be essential for prescribing behavioral therapy, including digital CBTi, and determining the treatment’s effectiveness and patient adherence. We previously developed sleep prediction models intending to eliminate the need for keeping sleep diaries by predicting the next sleep using only information from wearable devices [2]. Three other studies have predicted future sleep using data automatically collected from wearable devices or smartphones [3–5]. Two of these used only physical activity data to predict sleep [4,5], and the other used physical activity and light exposure data but predicted subjective sleep quality [3]. In our previous study, we used actigraphy data, including physical activity and light exposure, to predict good sleep, defined as ≥90% sleep efficiency.

Our previously developed sleep prediction model has several limitations. First, even though we collected data from 109 people for 14 consecutive days, the model performance was not excellent due to the relatively small amount of data and significant data loss (38% of the total data) due to battery depletion and non-contact with the body, among other causes. Including sufficient data in prediction model development is known to improve the model’s accuracy [6,7]. In this study, we aimed to solve this problem by using commercially available wearable devices and sending data to the cloud in real time, which could prevent data loss.

Second, sleep predictions based on physical activity and light exposure alone are limited. Existing research suggests that psychological stress also has a significant impact on sleep [8,9]. Although psychological factors are known to have an important impact on sleep, they have not been included in previous models, which may explain the models’ limited performance. Thus, we added a new variable to the sleep prediction model that reflects psychological state, as measured by wearable devices. Heart rate variability (HRV) is a physiological indicator of psychological stress that has gained attention in sleep research [10,11]. As it has not yet been used in sleep prediction models, we decided to investigate whether using HRV as an indicator of psychological state related to sleep would improve the performance of our sleep prediction models.

Finally, we considered the length of the data analyzed for sleep prediction. Three previous studies that used wearable devices to predict sleep developed models based on 1-day data [3–5]. We previously compared a model based on 2-day data with a model based on 1-day data and found that the performance of the 2-day data model was better [2]. Sleep is neither fragmented nor independent on a day-to-day basis. Instead, it exists in a dynamic and interconnected relationship with continuous lifestyle habits and the surrounding environment. Changes in lifestyle habits and the environment can influence sleep over short periods of several days and also over longer spans lasting months [12]. Therefore, 1 or 2 days of data may not be sufficient to predict future sleep. We aimed to determine whether we could improve the performance of our models with longer periods of data.

The purpose of this study was to improve the performance of our sleep prediction models by increasing the amount of data, adding indicators of psychological stress to physical activity and light exposure, and adjusting the data length. We developed several sleep prediction models using different data sources (i.e. exclusively wearable device data, exclusively sleep diary data, and combined data from both sources), analysis methods (machine learning, deep learning, and classical logistic regression), and data lengths (1, 2, and 3 days).

## Materials and methods

### Participants

Healthy participants who denied having sleep disturbance were enrolled in this study. We recruited participants *via* advertisements posted at Severance Hospital and Yonsei University College of Medicine. The exclusion criteria were as follows: poorly controlled medical condition (i.e. a condition resulting in a need for hospitalization within the last 3 months), regular use of medication for sleep, any current or previous major psychiatric disorder (e.g. schizophrenia or a related psychotic disorder, bipolar disorder, major depressive disorder, anxiety disorder), and substance use disorder. We determined the presence of psychiatric disorders, including substance use disorder, using the structured clinical interview for DSM-IV [13]. We invited individuals who agreed to participate by providing written informed consent to a screening interview in which the researchers (EC and DY) collected baseline characteristics, including sex, age, height, weight, education level, smoking status, alcohol consumption, caffeine use, and current psychiatric disorder diagnoses. The researchers also obtained baseline sleep-related information from the participants through the Pittsburgh Sleep Quality Index and Insomnia Severity Index [14,15].

### Ethical considerations

This study was conducted according to the Declaration of Helsinki principles. The institutional review board of Severance Hospital, Yonsei University Health System, approved this study (nos. 4-2017-0384 and 1-2019-0038). All participants provided written informed consent. All the collected data was anonymized before analysis.

### Data collection

Our previous preliminary study was conducted using only the Actigraph [2]. For the current study, we switched to a consumer wearable device for clinical application; however, we had 33 participants (11.9% of the total enrolled population) wear both devices simultaneously to verify the compatibility of data obtained from the two devices (Supplementary Figure 1). We found that the data from the two devices were compatible, thus we used data from both the preliminary study and the current study in our analysis. The ActiGraph GT3X+^®^ is a triaxial accelerometer with a light sensor, that has been validated against polysomnography and other activity monitors in several studies [16,17]. The Galaxy Watch Active2^™^ (Samsung) can monitor physical activity, light exposure, and HRV. Recent studies on consumer wearable devices, including the Galaxy Watch, have assessed and validated their use for research in medical fields [18–21].

After the screening interview, we asked participants to wear a device (either the ActiGraph or Galaxy Watch) on their non-dominant wrist continuously for 14 days, except while washing or participating in strenuous activities that could damage the device. We exported and assessed the collected data through the ActiLife software (version 6.13.3, ActiGraph) for the ActiGraph or an in-house program for the Galaxy Watch. We gathered data from the ActiGraph after the end of the 14 days of the experiment, whereas the Galaxy Watch sent data to a cloud server in real time.

We used sleep diaries based on the international consensus format to assess in-bed time, sleep onset latency, the number of awakenings after sleep, sleep time, wake time, out-of-bed time, caffeine consumption (defined as >1 cup within 6 h before sleep), alcohol consumption (defined as >10 g within 6 h before sleep), and whether the participant took naps [22]. We encouraged the participants to record the time whenever they removed the wearable device.

### Wearable device data processing

We collected physical activity and light exposure data every 10 seconds from both devices. The raw data are generally comparable across accelerometer brands [23], and previous studies have validated the comparability between consumer wearable devices and the ActiGraph [20,24]. Physical activity data from an accelerometer can be estimated using vector magnitude, which allows measurement of the extent of movement in three-dimensional space. The vector magnitude can be calculated using the coordinate values on the x-, y-, and z-axis measured by a triaxial accelerometer. This incorporates the vertical axis (z-axis: up–down) as well as the longitudinal (y-axis: forward–backward) and lateral (x-axis: left–right) axes [25]. We calculated the vector magnitude using the positional data from the ActiGraph and the Galaxy Watch to measure physical activity. Both devices use a photodetector to detect the light of their surroundings in lux. Light exposure data were gathered from the participants who wore both devices for 14 days, and the results showed no significant difference in this data between the devices.

We referred to previous study that developed a sleep prediction model using three different devices [4]. Previous studies demonstrated that the Galaxy watch can gather data at a level comparable to that of the Actigraph for overground walking and daily physical activity in free-living conditions [20,24]. In addition, we analyzed physical activity data in various situation (sitting, walking, and running) and light exposure data in indoor/outdoor environment from randomly selected participants who wore both devices simultaneously.

We excluded physical activity data or light exposure data from the analysis if there were any incomplete measurements due to issues such as device malfunction, battery depletion, or obstruction of the light sensor by long sleeves. It is not entirely possible to distinguish between genuine participant inactivity and depletion of the device battery, or between participants being in a dark environment and obstruction of the light sensor by long sleeves. We attempted to differentiate as much as possible by excluding data if there was continuous non-measurement of physical activity for 30 minutes or light exposure for 3 minutes. These criteria are consistent with our previous research [2].

We also measured HRV, which is an indicator of several conditions which affects the autonomic nervous system, including psychological stress [26]. HRV can be measured by electrocardiogram, cardiac belt, Holter monitor, or consumer wearable devices with a pulse monitor [27]. In this study, we used the R-R interval (RRI), measured with photoplethysmography, a pulse monitor on the Galaxy Watch, for every 2 consecutive minutes within the 30 min before sleep to calculate pNN50, defined as the proportion of successive RRIs greater than 50 milliseconds divided by the total number of RRIs [28]. Among the possible HRV indicators, pNN50 is considered to effectively reflect psychological states of relaxation or anxiety by measuring parasympathetic activity [29,30]. The ActiGraph does not have photoplethysmography, thus we developed new prediction models with HRV data from only the Galaxy Watch users.

When assessing sleep, we converted 10-second epochs into 60-second epochs using the ActiLife software (version 6.13.3, ActiGraph) and Python 3.6.4 (Python Software Foundation), which is consensus when measuring sleep by actigraphy [31,32]. This reflects the relatively stable movement patterns that occur during sleep compared with the active movement patterns that occur during wakefulness [31]. We used the Cole–Kripke algorithm, which identifies sleep/wake status from the mean value of y-axis actigraphy data, to measure in-bed time, sleep time, and wake time for both devices [32,33]. We used manual scoring in addition to the algorithm to avoid underestimating sleep latency or waking after sleep onset based on the Actigraphy Scoring Hierarchy Manual [34]. Furthermore, we used sleep diaries to assess the times when the devices were not worn. We followed the Society of Behavioral Sleep Medicine guidelines during actigraphy data scoring [35].

### Target outcome

The target outcome for ‘good sleep’ was ≥90% sleep efficiency, which matches the sleep efficiency target of 90% for sleep restriction therapy in CBTi [36,37]. Based on the data from the wearable devices, this study defined and calculated sleep efficiency as the ratio of total sleep time (sleep time to wake time) to the total time spent in bed (in-bed time to out-of-bed time) [38].

### Statistical analysis

**Figure 1.**
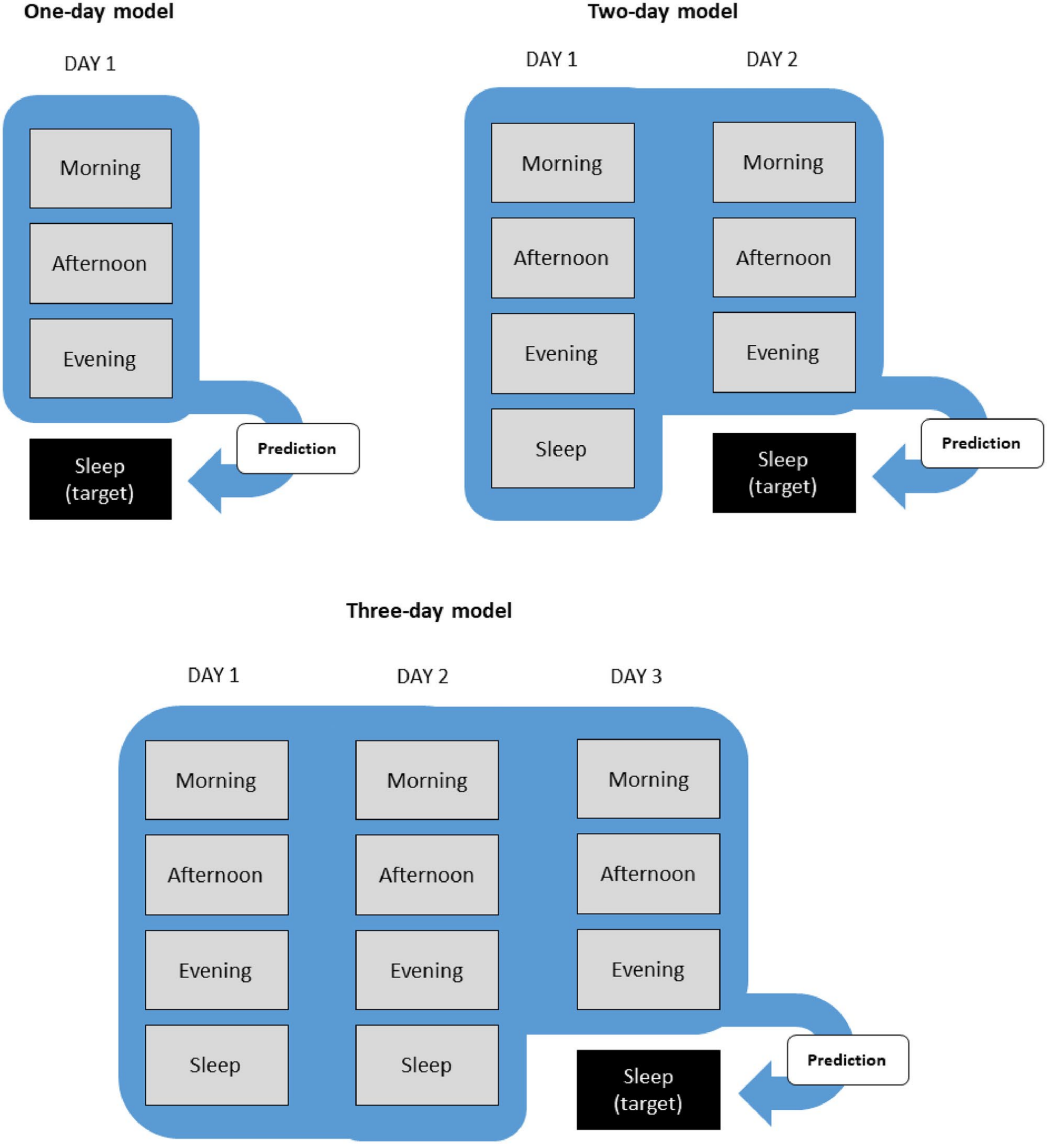
Data partitioning for the development of the sleep prediction models. Morning is the period from out-of-bed time to noon. Afternoon is the period from noon to 6 PM. Evening is the period from 6 PM to in-bed time. Sleep time indicates the period from in-bed time of the previous day to out-of-bed time of the same day.

In our previous study, we developed 24 different sleep prediction models with different data sources (wearable device, sleep diary, and combined data), data lengths (1-day and 2-day data), and analysis methods (machine learning, deep learning, and classical regression). We used extreme gradient boosting (XGBoost) for machine learning and convolutional neural network (CNN) and long short-term memory (LSTM) for deep learning. XGBoost is a scalable machine learning system for gradient tree boosting. It is based on a tree ensemble model, which combines decision trees to provide better predictive performance by creating more variance within the model [39]. CNN is a type of artificial neural network. Every layer of CNN transforms the input data to an output result of neuron activation, eventually leading to the final connected layers showing synthesized results [40]. LSTM is a recurrent neural network (RNN) model. RNN models are effective learning models for sequential data that use memory cells characterized by state maintenance over time [41]. All analysis methods were same as our previous preliminary study [2]. Here, we combined ActiGraph and Galaxy Watch data to create 24 sleep prediction models as before (Supplementary Figure 1). We developed prediction models with HRV and 3-day data from the Galaxy Watch data (Figure 1).

We measured model performance by accuracy and area under the curve (AUC). Accuracy referred to the number of correct predictions (both true positives and true negatives) divided by the total number of predictions. AUC values were used to show the combined predictability and validity of each model.

All statistical analyses were performed using R Studio version 1.1.463 (RStudio, Inc.) and Python 3.6.4 (Python Software Foundation).

## Results

### Characteristics of participants

**Figure 2.**
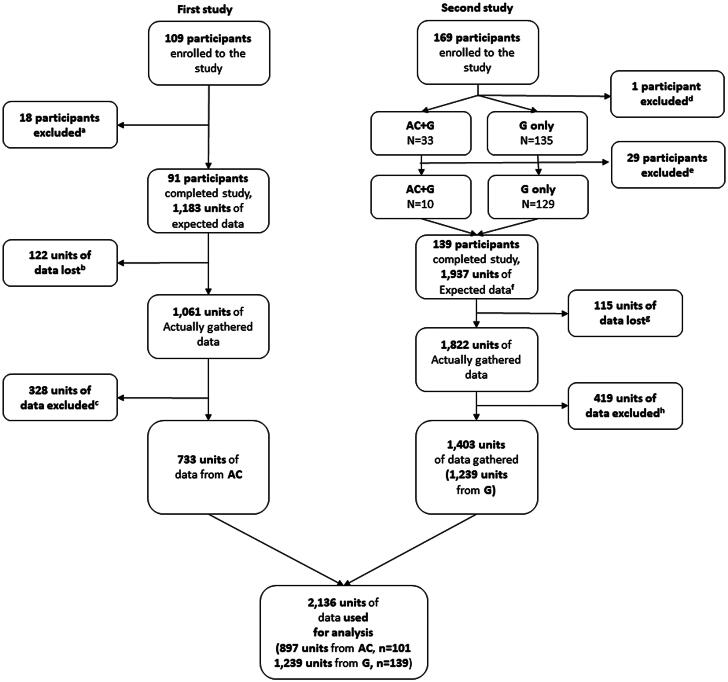
Flow diagram of participant enrollment and data selection. AC, ActiGraph; G, Galaxy Watch ^a^Consent withdrawal (n = 4), whole data lost (n = 3), incomplete data (n = 11); ^b^Battery depletion (n = 70), early study termination (n = 52); ^c^Lack of physical activity data more than 30 minutes (n = 109), lack of light exposure data more than 3 minutes (n = 219); ^d^Excluded due to narcolepsy; ^e^Whole data lost (n = 18), incomplete data (n = 11); ^f^13 units of data for AC (n = 10) and G (n = 139); ^g^Battery depletion (n = 62), early study termination (n = 53); ^h^Lack of physical activity data more than 30 minutes (n = 173), lack of light exposure data more than 3 minutes (n = 246).

Two hundred seventy-eight participants were enrolled in this study, including 91 of the 109 participants from the previous study who wore the ActiGraph [2]. The remaining 169 participants were newly recruited; one of these participants was screened out, 33 wore both the ActiGraph and Galaxy Watch at the same time, and 135 wore the Galaxy Watch only. Data from 29 participants were excluded from analysis (23 used both devices and 6 used the Galaxy Watch only) due to device errors (*n* = 18) or excessive time not wearing the device (*n* = 11). As 139 people completed the second 2-week study, the analysis ultimately included 2-week experiment data from a total of 230 participants (91 used the ActiGraph only, 129 used the Galaxy Watch only, and 10 used both devices). We defined one data unit as one day and one night of data; thus, because there were 13 possible data units per participant during the 2-week experiment, we could have theoretically collected 3120 units. The actual collected data included 2883 data units because 237 data units were lost during data collection (*n* = 132, battery depletion; *n* = 105, early study termination without consent withdrawal). We also excluded 282 data units related to physical activity and 465 data units related to light exposure due to insufficient measurements as described in the methods (Figure 2). Therefore, 2136 data units from 230 participants were used in the analysis. The analysis included data from 10 participants who wore both devices, 101 (897 data units) who wore only the ActiGraph, and 139 who wore only the Galaxy Watch (1239 data units). The 1-day sleep prediction models used 2136 units of data. For the 2-day prediction models, we combined two consecutive data units into a single unit, generating 1103 two-day units. The models based on consumer wearable device data were developed with 1239 data units for the 1-day model, 572 units for the 2-day model, and 308 units for the 3-day model.

The participant characteristics are presented in Table 1.

**Table 1.** Participant characteristics.

Variable	All participants (*n* = 230)	Participants with ActiGraph (*n* = 101)	Participants with Galaxy Watch (*n* = 139)	*P* value
Age, years, mean ± SD	33.5 ± 9.1	30.9 ± 8.8	35.5 ± 8.8	<.001
Sex, female, n (%)	146 (63.5)	65 (64.4)	90 (64.7)	.95
BMI, kg/m^2^, mean ± SD	22.6 ± 3.5	22.7 ± 3.6	22.6 ± 3.4	.93
Education, years, mean ± SD	16.9 ± 2.5	16.8 ± 1.9	16.9 ± 2.8	.77
Regular alcohol consumption, yes^a^, n (%)	142 (61.7)	56 (55.4)	92 (66.2)	.54
Current smoking, yes, n (%)	16 (7.0)	8 (7.9)	8 (5.8)	.28
Regular caffeine consumption, yes^b^, n (%)	192 (83.9)	81 (80.2)	122 (87.8)	.61
PSQI global score, mean ± SD	5.6 ± 2.6	5.9 ± 2.6	5.3 ± 2.6	.004
ISI, mean ± SD	6.1 ± 4.5	6.0 ± 4.2	6.3 ± 4.9	.55

Ten participants used both the Actigraph and Galaxy Watch.

PSQI, Pittsburgh Sleep Quality Index; ISI, Insomnia Severity Index.

^a^ >300 mL per week. ^b^>1 cup per day.

### Performance of the sleep prediction models

The performance of all the sleep prediction models (Tables 2 and 3; mean accuracy ± SD=.66±.00, area under the curve [AUC]=.69±.00) improved compared with the performance of the prediction models from our previous study (accuracy=.61±.00, AUC=.62±.01) [2]. The performance of the sleep prediction models with added HRV data (accuracy=.71±.01, AUC= .66±.01) also improved compared with the previous models. The 2-day models performed better (accuracy=.73±.06, AUC=.70±.08) than the 1-day models (accuracy=.68±.08, AUC=.64±.08) and the 3-day models (accuracy=.70±.07, AUC=.67±.08). The model with the best performance was the extreme gradient boosting model, XGBoost, with 2-day data units and combined actigraphy and sleep diary data (accuracy=.85, AUC=.80).

**Table 2.** Performance of sleep prediction models.

Analysis method	Parameter	Wearable device^a^		Sleep diary^b^		Combined^c^	
		1-day	2-day	1-day	2-day	1-day	2-day
XGBoost	Accuracy	.67	.66	.60	.62	.73	.74
	AUC	.69	.72	.61	.64	.77	.79
CNN	Accuracy	.63	.62	.65	.64	.68	.69
	AUC	.65	.57	.69	.68	.70	.75
LSTM	Accuracy	.63	.64	.58	.60	.69	.71
	AUC	.65	.68	.62	.62	.76	.77
Logistic regression	Accuracy	.62	.69	.64	.64	.69	.72
	AUC	.64	.66	.65	.66	.74	.74

XGBoost, extreme gradient boost; CNN, convolutional neural network; LSTM, long short-term memory; AUC, area under the curve.

^a^ Models developed from only wearable device data. ^b^Models developed from only sleep diary data. ^c^Models developed from both actigraphy and sleep diary data.

**Table 3.** Performance of sleep prediction models with the addition of heart rate variability.

Analysis method	Parameter	Wearable device^a^			Sleep diary^b^			Combined^c^		
		1-day	2-day	3-day	1-day	2-day	3-day	1-day	2-day	3-day
XGBoost	Accuracy	.60	.76	.70	.73	.74	.72	.82	.85	.85
	AUC	.52	.60	.57	.70	.68	.70	.75	.80	.79
CNN	Accuracy	.64	.59	.63	.62	.70	.61	.64	.72	.65
	AUC	.53	.55	.56	.58	.67	.60	.64	.70	.63
LSTM	Accuracy	.56	.76	.65	.71	.72	.72	.80	.83	.78
	AUC	.55	.60	.61	.70	.73	.71	.73	.77	.78
Logistic regression	Accuracy	.63	.68	.67	.69	.71	.69	.72	.73	.76
	AUC	.56	.62	.61	.68	.73	.71	.70	.77	.75

These models were developed from consumer wearable device (Galaxy Watch) data.

XGBoost, extreme gradient boost; CNN, convolutional neural network; LSTM, long short-term memory; AUC, area under the curve.

^a^ Models developed from only wearable device data. ^b^Models developed from only sleep diary data. ^c^Models developed from both actigraphy and sleep diary data.

## Discussion

This study aimed to improve sleep prediction models based on physical activity and light exposure by increasing the amount of data, adding a psychological stress indicator, and adjusting the data length. The XGBoost model developed from 2-day combined data and including HRV showed the best performance of all the models with 80% AUC and 85% accuracy. Although the 3-day data model did not outperform the 2-day model, this study proved for the second time that the existing sleep prediction model is valid and demonstrated its evolutionary potential.

It has been more than 20 years since actigraphy was first used in sleep research [42]. Unlike polysomnography, which usually measures sleep in a laboratory environment, actigraphy can evaluate sleep in everyday environments and can even monitor some activities during wake time. However, it cannot provide variables other than those approved by the manufacturers, and researchers cannot identify errors before the device is returned because the data are stored in the device. Furthermore, it is very expensive compared with consumer wearable devices, inevitably limiting the expansion of research results. To increase the amount of data and apply the results to patients with insomnia, a study using a popular consumer wearable device was needed. Sleep research using consumer wearable devices, including the Galaxy Watch, is becoming more common [43,44]. We conducted an experiment to compare data from a research device and data from a consumer wearable device, referring to previous studies that used two or more wearable devices to collect activity data [4,21,45,46]. We received Galaxy Watch data from the cloud in real time through the in-house program and tried to reduce data loss by monitoring the data uploads and contacting participants in case of errors. Although there was still significant data loss, we reduced the rate by 10% compared with previous studies, which may have improved the model performance. The availability of real-time data transmission and, therefore, the opportunity to reduce data loss are important developments that will allow sleep researchers to collect more data in the future.

We developed the models with HRV using only the data from the Galaxy Watch. Although the amount of data was consequently small (65% of the data volume of the models that used both ActiGraph data and Galaxy Watch data), the overall performance was better than that of the models without HRV regardless of the source, analysis method, or data length. HRV is used in sleep research because it reflects psychological arousal due to stress exposure. Estrela and colleagues found that lower high-frequency HRV (HF-HRV), reflecting increased sleep reactivity to stress, is associated with poor sleep quality [10]. MacNeil and colleagues also found that greater HF-HRV reactivity to worry correlates with sleep disturbance [11]. Instead of using HF-HRV, which is a frequency-domain index of HRV, we used pNN50, a time-domain index, to measure HRV. Both HF-HRV and pNN50 are known to reflect the activity of the parasympathetic nervous system, which in turn reflects stress reactivity [47]. The use of a time-domain indicator of HRV has the advantage of easy calculation; because pNN50 is calculated using RRI, resting HRV can be easily obtained from consumer wearable devices. HRV reflects changes in autonomic function, which means that it can be affected by various factors including age, sex, genetic factors, and medical conditions such as metabolic syndrome or pain [48–51]. Hence, HRV can be used as an objective assessment of stress and mental health, but it is important to consider other potential influencing factors [47]. In this study, we conducted research on healthy participants without significant psychiatric or medical history. Future studies including patients with various conditions can potentially improve the accuracy of sleep prediction. This is the first study to predict the quality of the next sleep by analyzing both physical and psychological state affecting sleep, and the results show that psychological state has a significant impact on the next future sleep.

We hypothesized that longer data collection periods would improve predictive power for the next sleep because we found that a 2-day model performed better than a 1-day model in our previous study. However, the results of the current study did not support this hypothesis. Previous studies that predicted future sleep with artificial intelligence used data from only a single day [3–5], so we can only speculate as to why the performance of our 3-day models was not better than that of the 1-day or 2-day models. Because many heterogeneous variables influence sleep, information about physical activity, light exposure, and HRV from more than two nights ago may not be enough to predict the next sleep. We assumed that the 2-day model performed better than the 1-day model because the 2-day data contained information about the previous night’s sleep; however, the information about the sleep from two nights before may have less of an impact on the next sleep prediction than the information from the previous night. Similarly, physical activity, light exposure, and psychological arousal from more than 2 days ago may have less of an effect on the next sleep. It is also possible that unmeasured variables may have long-term effect on sleep, potentially causing prediction errors. Another possible reason is that the 3-day models were based on a reduced amount of data compared with the 2-day and 1-day models because the data for the three consecutive days were grouped into one unit. To the best of our knowledge, no other studies have compared the effects of different data lengths on sleep prediction. Further studies are needed to verify the most efficient data length for sleep prediction.

This study has some limitations. First, the definition of ‘good sleep’ was not subjective but rather an objective measure of sleep efficiency. There has been controversy in clinical insomnia research over whether pharmacological/non-pharmacological treatment goals should be measured with objective indicators, such as sleep efficiency, or subjective sleep quality improvement [52]. This study’s definition of good sleep was based on sleep efficiency in consideration of CBTi, as improved sleep efficiency is often set as the goal of CBTi sleep restriction therapy and stimulation control therapy [53]. To apply the sleep prediction model developed in this study to CBTi in the future, additional validation in patients with insomnia is required. Second, although no participants reported sleep disturbances, 56.1% (*n* = 129) had a global Pittsburgh Sleep Quality Index score of at least 5 and 22.2% (*n* = 51) had a score of at least 8. A PSQI score above 5 generally indicates poor sleep quality, although some researchers have proposed that a cut-off score of 8 or higher is more appropriate to accurately screen for sleep problems [14,54]. The average PSQI score among our participants was high despite our recruitment of a healthy population of individuals who denied having sleep disturbance. This may be related to the fact that the researchers posted recruitment advertisements inside medical schools and hospitals. Among participants, 46.1% (*n* = 106) were medical practitioners or students, and individuals studying or working in medicine are often too busy to sleep. Lastly, the sleep prediction model was developed for participants who have not experienced sleep disturbances. Further research on insomnia patients is needed to demonstrate its clinical usefulness.

## Conclusion

Despite the above limitations, we have improved the performance of our previously developed sleep prediction models by increasing the amount of data and adding HRV as an indicator of psychological state. We expected improved sleep prediction by increasing the data length to 3 days, but the 3-day models failed to perform better than the 2-day models. We believe that these results, including the reliable performance of automated sleep prediction models and information about the factors that improve predictive performance, may provide a basis for further research in the field of non-pharmacological treatment for insomnia.

## Supplementary Material

Supplemental Material

## Data Availability

The data that support the findings of this study are available on request from the corresponding author. The data are not publicly available due to privacy or ethical restrictions.
