# Supplementary material for: Predicting sleep based on physical activity, light exposure, and Heart rate variability data using wearable devices
Source: Ann Med. 2024 Sep 19;56(1):2405077. doi: 10.1080/07853890.2024.2405077 (PMC11413959; doi:10.1080/07853890.2024.2405077)
Supplement: Supplemental Material [file IANN_A_2405077_SM4628.docx]

**Supplementary Figure 1. Schematic diagram of predictive model development in preliminary and the main study**

**
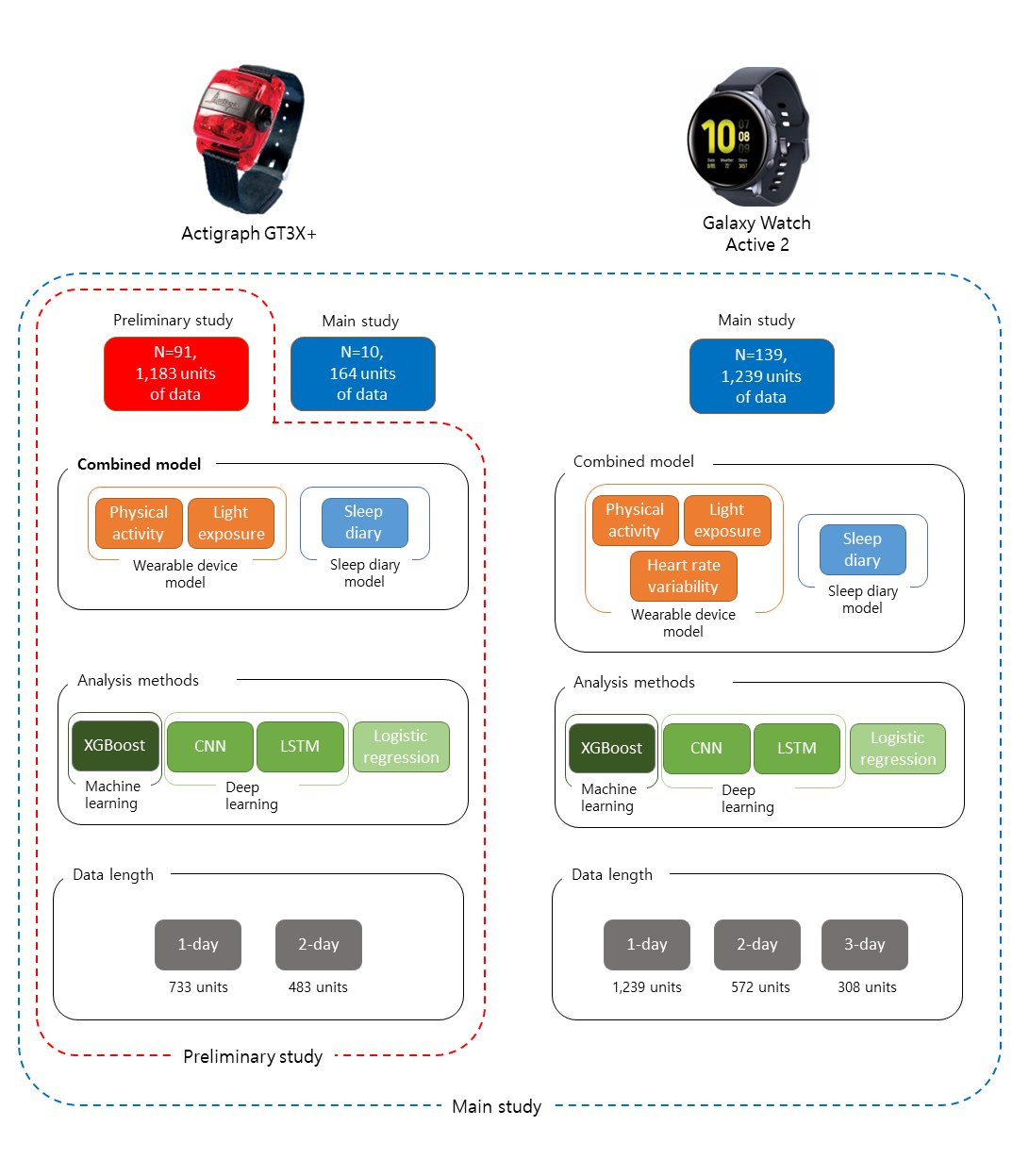
**

XGBoost, extreme gradient boost; CNN, convolutional neural network; LSTM, long short-term memory
